# Quantitative analysis of serum cell-free DNA as a predictive and prognostic marker in breast cancer patients

**DOI:** 10.3389/fonc.2023.1171412

**Published:** 2023-06-22

**Authors:** Gazi Nurun Nahar Sultana, Ferdowsi Akter, S. M. Hasan Israfil, Utpal Chandra Ray, Rumana Akther Jahan, Mohammad Shawkat Ali, Salim Al Din, Shafiqur Rahman, Rezaul Halim, Mohammad Sahajadul Alam

**Affiliations:** ^1^ Centre for Advanced Research in Sciences (CARS), University of Dhaka, Dhaka, Bangladesh; ^2^ Department of Clinical Pharmacy and Pharmacology, University of Dhaka, Dhaka, Bangladesh; ^3^ Department of Pharmaceutical Chemistry, University of Dhaka, Dhaka, Bangladesh; ^4^ Genetic and Cytology Laboratory, Invent Technologies, Banani, Dhaka, Bangladesh; ^5^ Institute of Statistical Research and Training, University of Dhaka, Dhaka, Bangladesh; ^6^ Department of Surgical Oncology, National Institute of Cancer Research and Hospital, Dhaka, Bangladesh

**Keywords:** breast cancer, cell-free DNA, biomarker, RT-qPCR, fluorometer, spectrophotometer

## Abstract

**Introduction:**

According to the GLOBOCAN (Global Cancer Observatory) 2020 report, 13,028 new cases of breast cancer (19%) were diagnosed in the United States, and 6,783 of them succumbed to the disease, making it the most common cancer among women. The clinical stage at the time of diagnosis is one of the most significant survival predictors in breast cancer. With delayed illness detection comes a lower survival rate. The prognosis of breast cancer may be predicted using circulating cell-free DNA (cfDNA), a non-invasive diagnosis technique.

**Objective:**

This study aimed to determine the most sensitive and effective method for detecting changes in cfDNA levels and for using cfDNA as a diagnostic and prognostic marker of breast cancer.

**Methods:**

The potential function of serum cfDNA levels as a marker for early breast cancer diagnosis was investigated using UV spectrophotometric, fluorometric, and real-time qPCR assays.

**Results:**

This research suggests that the most successful way to measure the amount of cfDNA described decades ago could be used as a "liquid biopsy" to track cancer in real time. The RT-qPCR (ALU115) method produced the most statistically significant results (p=0.000). At the threshold concentration of 395.65 ng/ml of cfDNA, the ROC curve reflects the maximum AUC= 0.7607, with a sensitivity of 0.65 and specificity of 0.80.

**Conclusion:**

For a preliminary assessment of total circulating cfDNA, a combination of all of the above techniques will be most efficacious. Based on our results, we conclude that the RT-qPCR technique combined with fluorometric measurement can identify a statistically significant difference in cfDNA levels between cohorts of breast cancer patients and healthy controls.

## Introduction

By the end of 2020, breast cancer had become the most common cancer in the world, with 7.8 million women surviving who had been diagnosed in the previous five years (1). Worldwide, Breast cancer takes more Disability Adjusted Life Years (DALYs) from women’s lives than any other malignancy. In 2020, breast cancer ranked first in incidence and mortality in most of the world’s countries (2). In Bangladesh, the circumstances are comparable. According to the GLOBOCAN (Global Cancer Observatory) 2020 report, 13,028 new cases of breast cancer (19%) were diagnosed in the United States, and 6,783 of them succumbed to the disease, making it the most common cancer among women (3). The clinical stage at the time of diagnosis is one of the most significant survival predictors in breast cancer. With delayed illness detection comes a lower survival rate (4). Therefore, if the disease is discovered early enough, we can minimize mortality, as breast cancer treatment at an early stage may be rather beneficial. So, screening for molecular markers as a way to find breast cancer early and treat the disease is one of the most promising areas of research (5).

Currently, there are several methods for the clinical diagnosis of breast cancer. Some are invasive, while others are non-invasive. Mammography is one of the most widely used non-invasive techniques for the early detection of breast cancer. However, its efficacy and precision have long been disputed due to its inability to detect small tumors, particularly in women with dense breast tissue (6). CA 15-3 is used to evaluate the response to invasive breast cancer treatment and identify disease recurrence. Because CA 15-3 is not very specific, it cannot be used as a standard breast cancer test for women (7). Monitoring metastatic stage 4 breast cancer and, in extremely rare cases, detecting recurrence in patients who have had treatment for early-stage breast cancer, are the two most prevalent uses of the CA 27.29 test (8). Metastatic breast cancer may be indicated by a high level of CA125. Due to the lack of clarity between CA125 levels and cancer, the test was not useful as a screening tool (9). The carcinoembryonic antigen (CEA) test is not a practical breast cancer screening tool; rather, it is utilized to determine cancer prognosis, treatment outcome, and recurrence. Due to its low sensitivity and specificity, the American Society of Clinical Oncology currently advises against using CEA for routine breast cancer screening (10). This shows that these variables are largely utilized to evaluate therapy success in advanced breast cancer patients. None of them have been used as a sole screening method because they are not very good at diagnosing or predicting early illness (11, 12). Studies have demonstrated that no combination of the 10 chosen serum breast cancer indicators, including CA15-3, CA125, and CEA, can properly diagnose cancer in its early stage. This is despite the fact that the combination of some of these biomarkers can boost sensitivity and specificity (5, 13, 14).

In order to overcome the drawbacks of these biomarkers in the early identification of breast cancer, there is an urgent clinical need for a simple and practical indicator. In the search for a biomarker, there has been a significant amount of interest in cell-free DNA, often known as cfDNA. The abbreviation “cell-free DNA” (cfDNA) refers to the small DNA fragments that may be detected in the circulation as well as other body fluids, including spinal fluid and urine. These fragments are obtained from a variety of sources, including the breakdown of cells, the release of DNA from damaged or dying cells, and the shedding of DNA by normal cells. Specifically, the release of DNA from damaged or dying cells is the most common source. The presence of cancer-associated DNA (cfDNA) in bodily fluids is an indicator of several processes, some of which include inflammation, physical damage, and cancer. One application of cfDNA in cancer diagnosis is as a liquid biopsy. A liquid biopsy involves analyzing the DNA fragments in a patient’s blood to detect the presence of cancer, without the need for a traditional tissue biopsy. This can be particularly useful in cases where it is difficult or risky to obtain a tissue sample, such as in some lung cancers or brain tumors. Liquid biopsies can also be used to monitor tumor progression and treatment response over time.

In terms of treatment, cfDNA can be used to identify genetic mutations or alterations that are specific to a patient’s tumor. This information can be used to guide targeted therapies, such as drugs that are designed to inhibit specific molecular pathways that are driving the growth of the cancer.

The correlation between cfDNA levels and clinical prognosis in cancer is an area of active research. Several studies have suggested that higher levels of cfDNA in cancer patients are associated with poorer outcomes, such as increased risk of disease progression and reduced overall survival. However, the relationship between cfDNA levels and prognosis can be complex and varies depending on the type and stage of cancer. Further research is needed to better understand how cfDNA can be used to predict clinical outcomes in cancer patients. The relationship between cfDNA expression and clinical and pathological features in breast cancer is an active area of research. Some studies have suggested that higher levels of cfDNA in breast cancer patients may be associated with more aggressive disease characteristics, such as larger tumor size, higher tumor grade, and more advanced stage (13–16). However, there have been few studies on the levels of cfDNA in Bangladeshi breast cancer patients. This study intends to examine the efficacy of cfDNA as a marker for early breast cancer detection and prognosis. The measurements were conducted using spectrophotometry (NanoDrop), fluorometric measurements (Quantus), and real-time qPCR. These three-way measuring procedures aided us in determining the best methodology for quantifying cfDNA as well as the inter-technique variation in results.

## Materials and methods

### Patients

From 2019 to 2020, 80 people were enrolled at the National Institute of Cancer Research and Hospital (NICRH) in Mohakhali, Dhaka. Of these, 69 had invasive breast cancer, 7 had infiltrative breast cancer, 2 had metastases, and 2 had unclear cancer types. The average life expectancy of breast cancer patients was 45.1 ± 8.775 years, while the average age of healthy people in the study was 39.14754 ± 13.233 years. The selected patients were required to complete an informed consent form in order to participate in the research project. Overview of clinical and pathological features of 80 breast cancer patients is shown in **Table 1**. The University of Dhaka’s Ethical Committee (Faculty of Biological Sciences) gave permission for the study to go ahead.

**Table 1:** Overview of clinical and pathological features of 80 patients.

Variables	Percentage
**Age (Mean)**	±45.10 Years
Marital Status	
Single	1.3%
Married	98.8%
Menstrual Cycle	
Postmenopausal	50.0%
Regular	47.5%
Irregular	2.5%
Comorbidities (Multiple Response)	
Uterus Complication	15.5%
Allergy	24%
Hypertension	42.5%
Diabetes Mellitus	38.5%
Hypothyroidism	18.6%
Type of Cancer	
Inf. DCC	20%
Inv. DCC	88.3%
Meta. DCC	2.5%
N/A	2.5%
Cell differentiation	
M.D	70%
P.D	11.3%
N/A	18.8%

N/A, Not Applicable.

### Serum separation

Serum was separated from whole blood using a two-step, progressive centrifugation technique. Blood was centrifuged for 10 minutes at 2500 rpm at room temperature in order to separate red and white blood cells. The transfer of WBCs was stopped by centrifuging the secondary serum at 1000 rpm for 10 minutes at 4˚C. Each patient had 3 ml of blood drawn and placed in an EDTA-coated vacutainer, from which the serum was extracted using a pipette so as not to damage the coating. The supernatant was collected using a new tube.

### cfDNA extraction

Following the manufacturer’s instructions, cfDNA was extracted from serum using the Maxwell^®^ RSC ccfDNA Plasma Kit (Madison, Wisconsin, USA). After adding 1 mL of serum samples to the prepared cartridges, the equipment was activated.

### Quantification by NanoDropTM 2000 spectrophotometer

Spectrophotometry is used to quantify chemicals in several scientific fields, including chemistry, physics, biochemistry, and therapeutic situations. Using a UV-Vis spectrophotometer called the NanoDropTM 2000 Spectrophotometer (Thermo Fisher Scientific, USA) for 1 µL of extracted cfDNA sample, the concentration of cfDNA was measured.

### Quantification by Quantus™ fluorometer

For the purpose of quantifying nucleic acids using Promega Quanti-Fluor^®^ Dye Systems (QuantiFluor^®^ dsDNA, Systems), the QuantusTM Fluorometer comes with its own set of settings already pre-programmed. By following the manufacturer’s instructions, it was possible to get an accurate measurement of cfDNA. For the purpose of calibrating the fluorometer, both blank and standard samples were generated. In contrast to the NanoDropTM, the Quantus fluorometer is based on the principle of fluorometry, which includes the use of very sensitive and precise fluorescent dyes to measure DNA, RNA, and protein. The UV-induced fluorescence of intercalating dyes is a more accurate and sensitive approach for detecting DNA than spectrophotometers. Since the intercalating dye only sticks to double-stranded DNA, it has no effect on proteins or ribonucleic acid molecules that are contaminating the DNA.

### Quantification by real-time quantitative polymerase chain reaction

As mentioned above, a short segment (115 bp) and a long fragment (247 bp) from a consensus sequence with multiple genomic ALU repeats were amplified and looked at to figure out how much serum cfDNA was there and how well it was put together (17). Short (apoptotic) and long (non-apoptotic) DNA fragments were amplified with the ALU115-bp primer, whereas long nonapoptotic DNA fragments were amplified with the ALU247-bp primer. The total quantity of serum DNA was determined using ALU115 primers by RT-qPCR technique.

For a 306-bp amplicon, the primer sequences were as follows: ALU115–forward, 5’–CCTGAGGTCAGGAGTTCGAG-3’ and reverse, 5’-CCCGAGTAGCTGGGATTACA-3’; ALU247–forward, 5-GTGGCTCACGCCTGTAATC-3’ and reverse, 5’-CAGGCTGGAGTGCAGTGG-3’. Standard curve concentrations were determined by serial 10-fold dilutions of female genomic DNA purchased from the Promega Corporation. The RT-qPCR (Real-Time Polymerase Chain Reaction) was conducted using a qTOWER3 from Analytik Jena (Thuringia, Germany). Each PCR reaction included 1 mL of cfDNA extract, 1 mL of forward primer, 1 mL of reverse primer, 7 mL of nuclease-free water, and 10 mL of the master mix (Promega GoTaq^®^ qPCR Master Mix). The DNA was replaced with water at a 1:1 ratio in the blank samples.

### Statistical analysis

SPSS Statistics 17.0 was applied for statistical analysis (SPSS Inc., Chicago, IL, USA). The statistically significant *P*-value utilized was 0.05. The Mann-Whitney U test was used to compare the cfDNA levels of people with breast cancer to those of healthy people to see if there was a statistically significant difference. The dispersion of concentration levels was also analyzed by calculating the interquartile range. The Kruskal-Wallis rank sum test was utilized to analyze the cfDNA level distribution difference between two research groups. The receiver operator characteristics (ROC) curve was designed to test the ability of serum cfDNA levels to discriminate patients from controls (**Table 2**). For the purpose of calculating sensitivity and specificity, each serum cfDNA concentration was used as a threshold value to determine the area under the curve (AUC).

**Table 2:** Statistical analysis of cfDNA level measured by Spectrophotometry, fluorometry, and RT-qPCR methods.

Methods	Health Status	Median	IQR	N	p-value (Median test)	p-value (MW test)
NanoDrop^TM^ Spectrophotometer	Healthy	900	1400	61	0.411	0.3496
	Breast cancer	1100	1200	80		
Quantus^TM^ Fluorometer	Healthy	440	252	61	0.002	0.0002
	Breast cancer	610	461	80		
RT-qPCR Alu-115	Healthy	216.2922	199.8659	61	0.000	0.0000
	Breast cancer	613.6191	875.9712	80		
Integrity Index (Alu247/115)	Healthy	1.5499	0.7249	61	0.072	0.0449
	Breast cancer	2.4239	5.970	80		

## Results

### cfDNA analysis obtained by spectrophotometry

The concentration of serum cfDNA was determined spectrophotometrically in order to evaluate the potential function of plasma cfDNA level as a marker for differentiating patients with breast cancer from healthy individuals. According to the NanoDropTM Spectrophotometer, the median concentration of cfDNA in breast cancer patients was 1100 ng/ml (IQR: 1400), while the median concentration in healthy persons was 900 ng/ml (IQR: 1200) (p = 0.411). Using the Mann-Whitney U test, the total concentration difference between the two research groups was determined to be p = 0.3496 (**Table 3**). Using the spectrophotometer to quantify cfDNA, the AUC for distinguishing breast cancer patients from healthy controls was found 0.5460 (95 percent confidence interval = 0.44767–0.64434). At a cutoff score of 600 ng/ml, the highest accuracy was achieved, with a sensitivity of 75%, a specificity of 41%, and positive and negative predictive values of 79.9% and 34.4%, respectively (**Table 2**).

**Table 3:** Cut-off value determination for three different techniques/Test performance characteristics in relation to various threshold values.

Methods	Area-ROC	(95% CI)	Cut off value (ng/ml)	Sensitivity (at the cutoff value)	Specificity (at the cutoff value)	Positive predictive value (%) (at the cutoff value)	Negative predictive value (%) (at the cutoff value)
NanoDrop^TM^ Spectrophotometer	0.5460	0.44767	600	0.75	0.41	79.9	34.4
		0.64434					
Quantus^TM^ Fluorometer	0.6818	0.59384	517	0.64	0.66	70.2	59.4
		0.76968					
RT-qPCR Alu-115	0.7607	0.68175	396	0.65	0.80	71.1	75.2
		0.83956					
Integrity Index (Alu247/115)	0.5988	0.50079	2.221	0.53	0.87	59.9	83.5
		0.69675					

### cfDNA analysis obtained by fluorometry

On the basis of fluorometry, the median concentration of cfDNA in healthy individuals and breast cancer patients was determined to be 440 ng/ml (IQR 252) and 610 ng/ml (IQR 461), respectively (p = 0.002), which was statistically significant. The Mann-Whitney U test revealed that the difference in total concentration between the two study groups was p = 0.0002. Using the fluorometer, the AUC for distinguishing breast cancer patients from normal controls was 0.6818 (95% confidence interval [CI] = 0.59384-0.76918). At a cutoff value of 517ng/ml, the maximum accuracy was attained, with 64% sensitivity and 66% specificity; the positive and negative predictive values were 70.2% and 59.4%, respectively.

### cfDNA analysis obtained by real-time quantitative polymerase chain reaction

**Figure 1:**
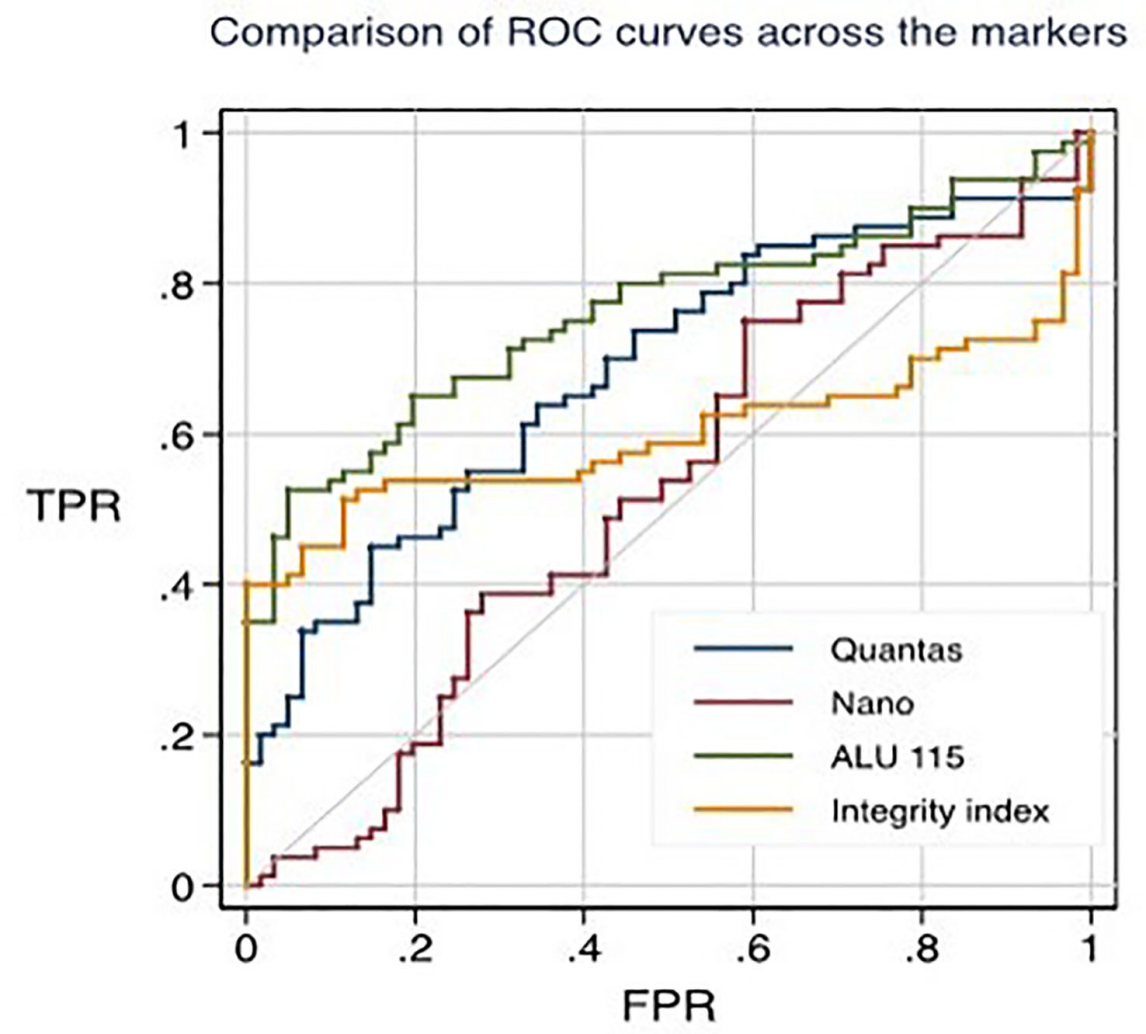
ROC curves for distinguishing healthy individuals from primary breast cancer patients through different techniques.

Using the RT-qPCR method, the researchers found that the median cfDNA level for ALU115 in healthy participants was 216.2922 ng/ml (IQR: 199.8659), while it was 613.6191 ng/ml (IQR: 875.9712) in breast cancer patients (p = 0.000). Using the Mann-Whitney U test, it was determined that the difference in concentration between the two study groups was statistically significant (p = 0.0000). For separating breast cancer patients from healthy controls, ALU-115 had an AUC of 0.7607 (95% CI = 0.68175–0.83956) (**Figure 1**). At a cut-off value of 395.6511 ng/ml, there was the most accuracy, with a sensitivity of 65% and a specificity of 80%. The positive predictive value was 71.1%, and the negative predictive value was 75.2%.

### cfDNA integrity index

**Figure 2:**
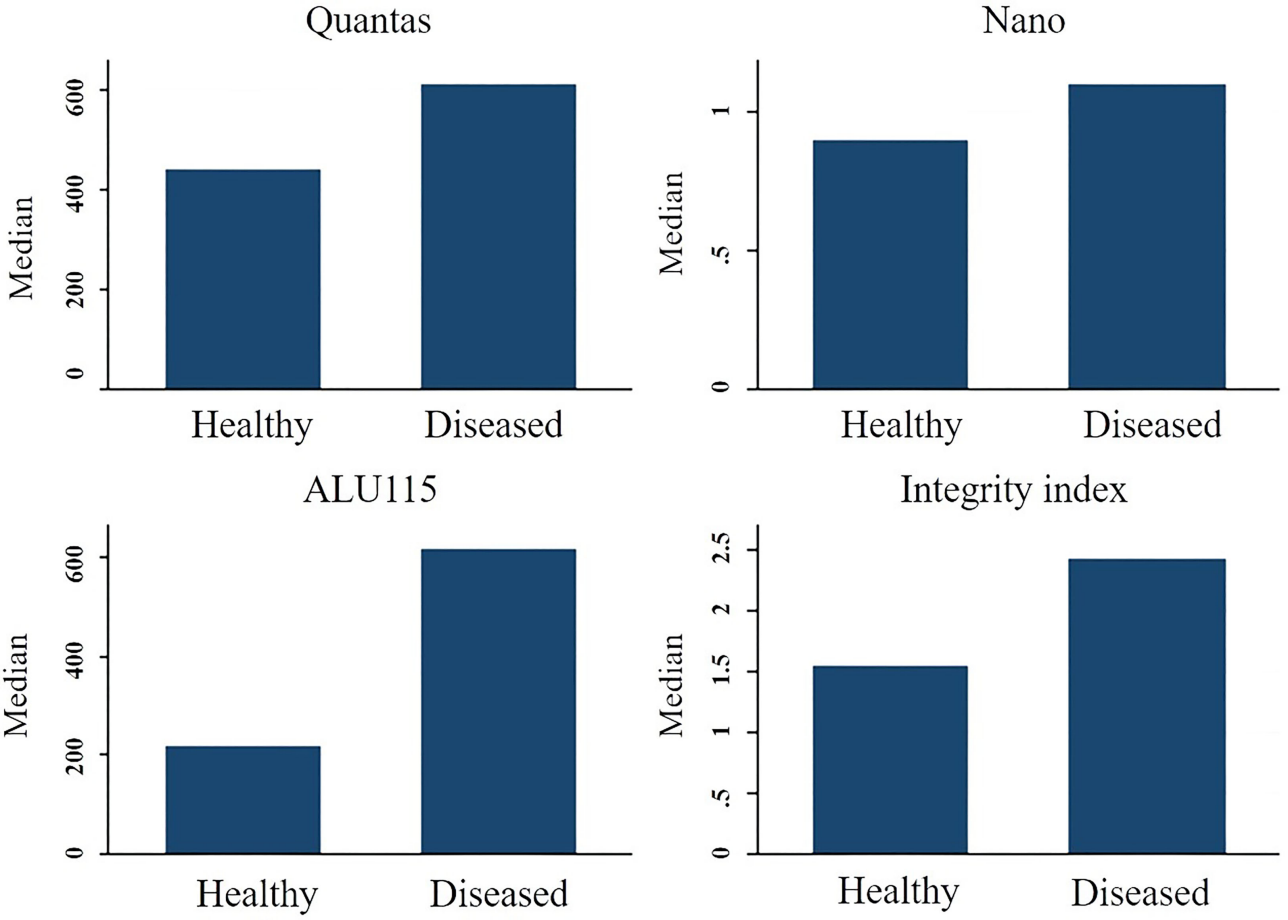
The median cfDNA concentration difference between healthy people and breast cancer patients using various techniques.

The median integrity of cfDNA (247 bp/115 bp ratio) for healthy and breast cancer patients was found 1.5499 (IQR-0.7249) and 2.4239 (IQR-5.970) respectively (p = 0.072). Mann-Whitney U test showed the overall concentration difference between the two study groups was p = 0.0449. The AUC for distinguishing breast cancer patients from normal controls by measuring cfDNA integrity was 0.5988 (95% CI = 0.50079-0.69675). The maximum accuracy was achieved by using a cut-off value of 2.221, which had a sensitivity of 53% and a specificity of 87%. The positive predictive value was 59.9%, whereas the negative predictive value was 83.5% (**Figure 2**).

This study compared the cut-off values of cfDNA with other studies. **Table 4** shows the comparison references. Bangladesh, India (27), and China (28–30) shows consistent cut-off values of cfDNA. Other study of USA (31), UK (32), Portugal (29) shows very low cut-off value of cfDNA. However other study of India (33) and Isreal (34) show high values.

**Table 4:** Comparison of cut-off-values from different countries.

Country/Year	Samples	Assay Methods	Cut-off Value (ng/mL)	Reference
Bangladesh/ 2022	Serum	RT-qPCR	397	Current study
India/2015	Plasma	RIA	600	Zaher et al (18)
India/2022	Serum	RT-qPCR	266	Kumari et al (19)
USA/1977	Serum	RIA	50	Leon et al (20)
UK/2004	Serum	RT-qPCR	100	Gal et al (21)
China/2012	Plasma	RT-qPCR	220	Huang et al (22)
Portugal/2012	Plasma	RT-qPCR	106	Catarino et al (23)
China/2012	Serum	RT-qPCR	471	Gong et al (24)
Israel/2015	Serum	FSGS	600	Agassi et al (25)
Thailand/2015	Plasma	RT-qPCR	100	Tangvarasittichai et al (26)

## Discussion

The evaluation of cfDNA using a variety of approaches is vital; thus, the development of a simple, economical, and standardized procedure is a significant step towards its deployment and broad use. This study used three different methods (spectrophotometry, fluorometry, and RT-qPCR) to look at how serum cfDNA level could be used as a biomarker to tell the difference between breast cancer patients and healthy people. Additionally, RT-qPCR is employed to determine the integrity index of cfDNA. According to our knowledge, this is the first study of its sort to determine the level of cfDNA in breast cancer patients and healthy individuals in Bangladesh. Our objective was to design a blood-based biomarker that was both inexpensive and simple to detect. As a result, the sequence non-specific NanoDropTM spectrophotometer and Quantus Fluorometer, both of which are user-friendly and inexpensive. Sequence-specific RT-qPCR, the gold standard for quantifying cfDNA, was applied alongside both techniques.

Identification of cancer at an early stage is absolutely necessary for therapy to be successful. In a significant number of instances, the most up-to-date screening techniques are unable to detect breast cancer in its early stages. It has been demonstrated that the plasma/serum cfDNA integrity index rises with the progression of a variety of cancers and among them, cancer of the breast is one. Most research used ALU quantitative polymerase chain reaction (PCR) to figure out the DNA integrity index (DII), which is the ratio of ALU247 long fragments made by dead cells to ALU115 short fragments made by healthy cells (33). The cfDNA integrity index (II), which has been proposed as a potential cancer marker (16). The ratio of RT-qPCR results was used to calculate the cfDNA integrity index (ALU247 and ALU115). Some studies have quantified total cfDNA levels using GAPDH, beta-globin, beta2-microglobulin, hTERT, or LINE-1 as possible target genes, employing greater cfDNA levels to differentiate benign from malignant BC (27–29, 31, 32). The detection of ctDNA levels through the use of the cell-free DNA integrity (cfDI) measurement, is more specific than the measurement of total serum cfDNA. This method has been investigated in BC through the use of qPCR by a number of authors with the SYBR Green fluorescent dye (30). Normal cells release DNA fragments of different sizes when their nucleosomes are broken apart by enzymes during the apoptosis process. On the other hand, tumor cells die in many different ways, such as necrosis and autophagy, and also release DNA fragments of different sizes (34, 35). Using ALU targets, Umetani et al. (36) presented cfDI as a helpful technique to detect main BC, demonstrating that it could be used to characterize lymph node metastases in a cohort of 83 patients compared to 51 healthy controls. They measured in blood shorter fragments of 115 bp thought to come from apoptotic normal cells and larger fragments of 247 bp thought to come from cancer cells necrosis or autophagy.

All approaches revealed a greater amount of cfDNA in breast cancer patients compared to healthy individuals. The RT-qPCR (ALU115) technique yielded the most significant results. Consistent with previous research findings, the Mann-Whitney U test of the RT-qPCR data demonstrated a significant difference in cfDNA concentration between the two study groups (P = 0.0000) (34). The fluorometric method distinguished between healthy individuals and those with breast cancer (p = 0.002). Additionally, the Mann-Whitney U test’s P value showed a statistically significant difference (P = 0.0002) between the two groups. The results were also consistent across other investigations (18, 19). Using research from the past, real-time PCR was used to multiply ALU 115-bp and 247-bp sequences inside ALU repetitions (19–21). More than 10% of the human genome is made up of ALU sequences; they are the most common and active repeating elements, and their typical length is under 300 nucleotides (22, 36). Fluorometric and ALU RT-qPCR measures have more discriminatory power than spectrophotometric and Integrity index assessments of cfDNA level. The NanoDropTM spectrophotometric approach has a P value of 0.411, whereas the II measurement had a P value of 0.072. The Mann-Whitney U test’s P value indicated slightly more discriminating power. The NanoDropTM spectrophotometer reading was 0.3496, whereas the II measurement was 0.0449. Contrary to our findings, numerous studies (23, 34) have discovered a significant difference in cfDNA level integrity between healthy individuals and breast cancer patients using ALU repeats (19–21, 23). Compared to other methods, the spectrophotometric method in our study overestimated cfDNA. This is similar to what scientists found when they used the NanoDrop method, which also overestimated cfDNA (24). NanoDrop is the most economical and time-efficient of the three methods, needing only a few seconds for each measurement and requiring minimal installation. So, we might be able to use it to check the purity of cfDNA samples right from the start.

The introduction of enormous new data from “omics” in cancer diagnosis will gradually alter cancer’s therapeutic and diagnostic approaches. Fragmentomics analysis, namely fragment size and cfDI, is gaining attraction in British Columbia as a non-invasive, cost-effective new technique of information gathering. It will specifically change the method to liquid biopsy, which will allow for the acquisition of useful information independent of the mutational signature. As a result of modern bioinformatic tools (i.e., DNA assessment of fragments for early interception, DELFI), epigenetic characteristics are more dynamic, and global fragmentation-patterns might be integrated as distinct biomarkers to indicate breast cancer.

This study indicated that the three test techniques produced significantly different quantities of cfDNA from the same sample in some instances. Others have noted these repercussions as well (24). It is necessary to overcome the discrepancy in their capacity to accurately measure distinct cfDNA fragment sizes.

In addition, qPCR findings demonstrated a 67 percent decrease in the concentration of DNA fragments with a size of 150 bp as compared to intact (unfragmented) genomic DNA (25). In contrast, fragmentation to 150 bp had no effect on the amount of DNA measured by the NanoDrop instrument, most likely because fragmentation has no effect on absorbance values (26).

UV spectroscopy cannot distinguish between double-stranded DNA (dsDNA), single-stranded DNA (ssDNA), RNA, and nucleotides because nucleic acids have the highest absorption. The fluorescence spectroscopic method, on the other hand, measures the amount of intact dsDNA. As DNA fragmentation and denaturation increase, so does the amount of intact dsDNA (37–40).

In this study, we compared the cutoff value for cfDNA in breast cancer patients with the results of other studies that were conducted on breast cancer populations (**Table 4**) (23, 25, 41–47). This study shows that consistent cutoff values may be used for patients with breast cancer in Bangladesh. The potential applications seem to be numerous, from cancer detection and the anticipation of recurrence and the evaluation of minimal residual disease, to responsiveness or resistance to treatments. However, the gap between research and clinical practice is still deep, and larger studies are needed to reveal additional study gaps.

## Conclusion

In conclusion, liquid biopsy has the potential to find new pathways for the detection and treatment of breast cancer. These new avenues might include genetic testing and immunotherapy. We propose a sequential combination of the NanoDrop, Quantus, and RT-qPCR methods for a preliminary evaluation of total circulating cfDNA that is both cost-effective and comprehensive. We want to underline that the RT-qPCR method and the fluorometric measurement have the ability to identify a substantial difference in the amount of cfDNA in breast cancer patient cohorts in comparison to healthy controls.

It’s possible that the diagnostic relevance of these prospective biomarkers might be limited if there is not a method that’s both better and more reliable for assessing cfDNA concentrations. It is imperative that extraction and quantification technologies be standardized and made more cost effective before any attempts are made to implement them into ordinary clinical and laboratory practice. Before speculating on the diagnostic utility of cfDNA, further study is required to better describe it as well as determine the causes and processes that modulate its amount in blood, both under normal settings and in the presence of illness. The use of cfDNA as a routine diagnostic process in clinical practice is fraught with difficulties, and the field of clinical use of cfDNA is still in its infancy. In order to further confirm the findings and offer more scientific data to support the practical adoption of liquid biopsy procedures in breast cancer, multi-center, prospective large-scale clinical studies are required.^1^

## Data availability statement

The original contributions presented in the study are included in the article/supplementary material. Further inquiries can be directed to the corresponding author.

## Ethics statement

The studies involving human participants were reviewed and approved by Ethical Review Committee, Faculty of Biological Sciences, University of Dhaka, Bangladesh. The patients/participants provided their written informed consent to participate in this study.

## Author contributions

Conceptualization: GS, RJ. Funding acquisition: GS, RJ. Methodology: FA, UR, SD, SI, RH. Supervision: GS, SA, MAli. Validation: GS, FA, UR, SI. Resources: Manuscript. Statistical analysis: GS, SR, FA. Writing: GS, FA. All authors have read and agreed to the published version of the manuscript. All authors contributed to the article and approved the submitted version.

## Footnotes

1 cfDNA, cell-free Deoxyribonucleotide; RT-qPCR, Real-time quantitative Polymerase Chain Reaction; ROC, Receiver operating characteristic curve; AUC, Area under the curve; ssDNA, single-stranded DNA; dsDNA, Double stranded DNA.
